# Editorial: Combination therapies in cancer treatment: enhancing efficacy and reducing resistance

**DOI:** 10.3389/fphar.2025.1770248

**Published:** 2026-01-07

**Authors:** Xinyu Wang, Milica Pešić, Ana Podolski-Renić

**Affiliations:** 1 Department of Biomedical Sciences, Philadelphia College of Osteopathic Medicine, Suwanee, GA, United States; 2 Department of Neurobiology, Institute for Biological Research “Siniša Stanković”- National Institute of Republic of Serbia, University of Belgrade, Belgrade, Serbia

**Keywords:** antibody-drug conjugates (ADC), combination therapies, DNA damage response (DDR), drug resistance, ferroptosis, immunotherapy, STING pathway

## 1 Introduction

Cancer’s complexity, including diverse cell types and drug resistance, hinders long-term patient survival. Single-agent therapies are often ineffective, prompting a move towards combination therapies that target multiple survival pathways in cancer cells. This strategy can prolong treatment responses, resensitize resistant tumors, and lower drug doses to reduce toxicity while preserving effectiveness.

This Research Topic includes 19 contributions on new pharmacological strategies for enhancing tumor elimination and minimizing resistance. It highlights the importance of rational, mechanism-based combinations adapted to each patient’s unique molecular and immunological profile for effective cancer control.

## 2 Advancing immunotherapy and T-cell engagement

Immunotherapy has revolutionized cancer treatment, and much research is focused on combining immune checkpoint inhibitors (ICI) with other modalities, such as chemotherapy and targeted therapy, to overcome primary and acquired resistance. The core principle of this strategy involves leveraging chemotherapy to induce immunogenic cell death, thereby releasing tumor antigens and danger signals that effectively prime the immune system for attack. This mechanistic foundation is detailed in the review Li et al., which synthesizes preclinical and clinical evidence supporting rational scheduling and dosing of chemotherapy–ICI combinations.

The clinical potential of such immune-oncology doublets is exemplified by the meta-analysis Tang and Zhou. This study found that combining PD-1/PD-L1 inhibitors with tyrosine kinase inhibitors significantly improved progression-free survival and objective response rate in patients with unresectable hepatocellular carcinoma, particularly among Asian patients and those infected with hepatitis B virus, without an unacceptable increase in severe adverse events. In a complementary real-world setting, Guo et al. showed that extending immunochemotherapy beyond first-line treatment can offer clinically meaningful benefit in recurrent small-cell lung cancer, highlighting how combination strategies may reshape treatment paradigms even in aggressive, traditionally chemo-sensitive malignancies.

A particularly notable contribution in this Research Topic is the work Choi et al. Soft-tissue sarcomas are prototypical “cold” tumors with sparse lymphocytic infiltrates and limited responsiveness to immunotherapy. By combining the DNA-damaging agent doxorubicin with deliberate activation of the STING (Stimulator of Interferon Genes) pathway, the authors demonstrate that chemotherapy-induced release of tumor DNA into the cytosol can be harnessed to trigger cGAS–STING signaling, type I interferon production, and enhanced recruitment and activation of effector immune cells. This dual-action approach not only amplifies the direct cytotoxicity of doxorubicin but also remodels the tumor microenvironment toward an inflamed, T cell–permissive state, providing a compelling blueprint for transforming immunologically “cold” sarcomas into “hot” tumors amenable to additional immunotherapeutic interventions.

Beyond systemic antibodies and innate immune modulators, innovative cell-based strategies aim to broaden and deepen antitumor immune responses. The article He et al. explored the vast potential of TCR-engineered T cells, which can recognize intracellularly derived peptide–MHC complexes and thus target a broader antigenic repertoire than chimeric antigen receptor T (CAR-T) cells. The review discusses combination strategies that integrate TCR-T cells with checkpoint inhibitors, oncolytic viruses, or targeted agents to overcome antigen heterogeneity, T-cell exhaustion, and immunosuppressive microenvironments. Complementing this immunoengineering perspective, Liu et al. introduced hybrid nanovesicles co-decorated with PD-1 and signaling regulatory protein alpha (SIRPα) receptors to simultaneously disrupt the “do not find me” (PD-1/PD-L1) and “do not eat me” (CD47/SIRPα) signals. In melanoma models, this combinatorial blockade translated into robust antitumor activity and illustrates how multi-receptor targeting on a single nanoplatform can synergistically amplify innate and adaptive immune responses.

## 3 Targeted DNA damage response (DDR) and cell cycle disruption

A second major theme centers on exploiting cancer-specific vulnerabilities in DNA repair and cell-cycle regulation. The review Qian et al. provides a comprehensive overview of how inhibitors of DNA-PK, ATM, ATR, Wee1 and other DDR kinases can be combined with DNA-damaging agents to induce synthetic lethality. The authors emphasize the importance of genomic and functional biomarkers, such as homologous recombination deficiency signatures, to select patients most likely to benefit from DDR inhibitor–based combinations and to avoid overlapping hematologic and gastrointestinal toxicities.

This conceptual framework is further strengthened by the data-driven clinical synthesis Fontenot et al. By systematically compiling clinical trial outcomes across tumor types, the review delineates where combinations of DDR inhibitors (DDRis) with DNA-damaging agents (DDAs) have achieved meaningful efficacy and where toxicity or lack of stratification has limited success. Together, these two reviews underscore that the future of DDR-based combination therapy lies in rational pairing of agents, careful dose optimization, and biomarker-guided patient selection.

Several mechanistic studies in this Research Topic provide concrete examples of these principles. In Li et al., inhibition of PCNA was shown to synergize with the PARP inhibitor olaparib by disrupting the PCNA/PARP1 axis, impairing DNA repair and suppressing proliferation and invasion of hepatocellular carcinoma cells. This work highlights a previously underappreciated node of vulnerability that could be exploited in combination with clinically available PARP inhibitors.

Similarly, Ma et al. investigated the role of the cyclin-dependent kinase CDK2 in radioresistance. The authors demonstrate that Milciclib induces G1 arrest, downregulates CDK2 and cyclin E1, and impairs Rad51-mediated DNA repair. When combined with ionizing radiation, Milciclib significantly enhances apoptosis and partially reverses radiation resistance in colorectal cancer cell lines, achieving significant sensitizer enhancement ratios in resistant models. These findings position CDK2 inhibition as a promising approach to resensitize tumors to radiotherapy and exemplify how modulation of cell-cycle checkpoints can be integrated with genotoxic therapies for maximal impact.

## 4 Combination of antibody- and toxin-based therapeutics

Antibody-based therapies, including antibody–drug conjugates (ADCs) and recombinant immunotoxins, leverage antigen specificity to improve the therapeutic index of potent cytotoxic payloads. The mini-review Shi et al. summarizes how pairing ADCs with immune checkpoint inhibitors, conventional chemotherapy (e.g., taxanes and platinum compounds), or targeted small molecules can overcome resistance mechanisms such as antigen heterogeneity, drug efflux, and adaptive signaling rewiring. The authors highlight emerging clinical evidence that such combinations can deepen responses and extend survival in breast, lung, and urothelial cancers, while underscoring the need for vigilant monitoring of overlapping myelosuppression and neuropathy.

In parallel, the review Rashad et al. focuses on recombinant immunotoxins, such as moxetumomab pasudotox and tagraxofusp, which fuse bacterial or plant-derived toxins to antibodies or cytokines that recognize hematologic malignancies. The article discusses combination strategies that incorporate these agents with chemotherapy or hypomethylating drugs to eradicate minimal residual disease, prevent antigen-negative relapse, and achieve deeper remissions in otherwise refractory leukemia and lymphoma.

Beyond protein–toxin fusions, innovative radiopharmaceutical combinations add another dimension to this theme. In Ling et al., the authors demonstrate that the radioisotope Iodine-131 not only causes classical radiogenic DNA damage but also triggers ferroptosis by downregulating the cystine transporter SLC7A11. When combined with the ferroptosis inducer sulfasalazine, Iodine-131 produces marked synergistic antitumor effects in thyroid cancer cells. This work illustrates how integrating radiotherapy with regulated cell-death modulators can expand the cytotoxic repertoire beyond apoptosis and necrosis.

## 5 Integration of traditional and natural agents

The incorporation of traditional medicine and naturally derived compounds into modern oncologic regimens provides a critical perspective on modulating both systemic and tumor-specific environments. The systematic review Chen et al. offers high-level evidence that adding Traditional Chinese Medicine formulations to transarterial chemoembolization (TACE) significantly improves overall response and disease control rates, prolongs overall survival, and reduces adverse events such as abdominal pain and nausea. Network pharmacology analyses further suggest that multi-component herbal preparations exert pleiotropic effects on angiogenesis, inflammation, and apoptosis, providing a mechanistic rationale for their combination with locoregional chemotherapy.

This concept of multi-target, low-toxicity modulation is also showed in the prospective cohort study Li et al. Here, the addition of Huaier granules to a backbone of targeted therapy plus immunotherapy significantly extended median progression-free survival in unresectable HCC without compromising safety, supporting the feasibility of integrating evidence-based traditional agents into complex systemic regimens.

Two research article contributions focus on natural products as chemosensitizers. Huseynova et al. shows that the flavonoid apigenin synergistically enhances the cytotoxic and pro-apoptotic effects of L-asparaginase in T-cell acute lymphoblastic leukemia cells. By modulating oxidative stress, mitochondrial integrity, and pro-survival signaling, apigenin may permit dose reduction of L-asparaginase and thereby alleviate its dose-limiting toxicities. In a solid tumor context, Shcherbakova et al. highlights that lichen-derived compounds such as evernic acid can restore temozolomide sensitivity in glioblastoma models, likely through modulation of the Wnt signaling pathway and drug efflux mechanisms. These studies collectively suggest that rational incorporation of phytochemicals into conventional chemotherapy regimens may offer a route to overcoming resistance while preserving or even improving tolerability.

## 6 Advanced technology and drug discovery platforms

Modern combination therapy development increasingly depends on innovative delivery platforms and computational tools. The review Zhou et al. emphasizes the growing role of local drug delivery systems in the postoperative setting. By implanting or spraying nanomedicine-loaded materials directly into the surgical bed, these approaches can achieve sustained, localized release of chemotherapeutic agents, immune modulators, photothermal and photodynamic sensitizers, and even CAR-T cells. Such strategies have the potential to eradicate residual tumor cells, prevent local recurrence and distant metastasis, reduce systemic toxicity, and simultaneously manage postoperative inflammation and wound healing.

Complementing these physical platforms, Kang et al. introduces a sophisticated deep learning framework to address the vast combinatorial search space inherent to multi-drug regimens. By integrating molecular fingerprints of drug pairs with gene-expression–based representations of cancer cell lines and using transformer-based feature aggregation with uncertainty calibration, CDFA outperforms previous machine-learning and deep-learning models in predicting synergistic combinations. This work exemplifies how data-driven, AI-assisted pipelines can prioritize the most promising drug pairs for experimental validation, thereby accelerating the discovery of clinically actionable combinations and reducing the cost and time associated with empirical high-throughput screening.

## 7 Conclusion

**FIGURE 1:**
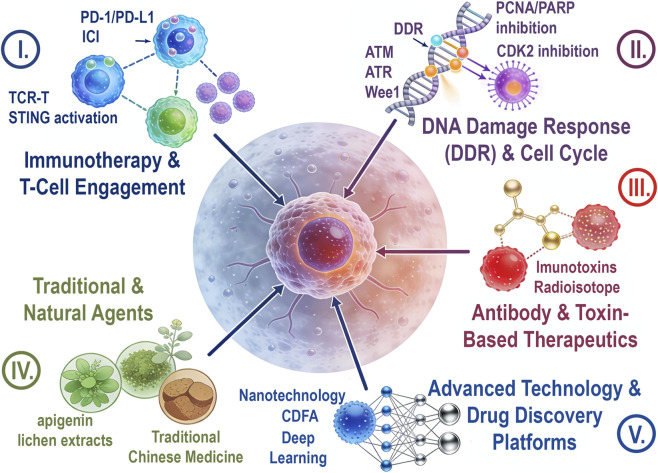
Diverse therapeutic strategies to enhance chemotherapy and radiotherapy, as well as innovative targeted and immunotherapy, presented in the Research Topic.

This Research Topic emphasizes the shift in oncology towards rational combination therapies. The 19 contributions explore topics such as targeting DDR, inducing ferroptosis, activating the STING pathway, and advancements in nanotechnology, cell therapy, and AI-based drug screening, along with clinical studies on complex treatment regimens (Figure 1). Overcoming resistance requires combining agents with complementary mechanisms and incorporating biomarkers and advanced delivery systems. Key themes include reshaping the tumor microenvironment for immune response, targeting multiple survival pathways, and balancing efficacy with toxicity using natural agents. These efforts aim for durable cancer control, supported by interdisciplinary collaboration and emerging technologies in precision medicine.

